# Ontogeny of Intestinal Epithelial Innate Immune Responses

**DOI:** 10.3389/fimmu.2014.00474

**Published:** 2014-10-09

**Authors:** Mathias W. Hornef, Marcus Fulde

**Affiliations:** ^1^Institute for Medical Microbiology and Hospital Epidemiology, Hannover Medical School, Hannover, Germany; ^2^Institute of Medical Microbiology, RWTH University, Aachen, Germany

**Keywords:** neonate, innate immunity, mucosal immunology, pattern recognition receptor, antimicrobial peptide, development, infection, inflammation

## Abstract

Emerging evidence indicates that processes during postnatal development might significantly influence the establishment of mucosal host-microbial homeostasis. Developmental and adaptive immunological processes but also environmental and microbial exposure early after birth might thus affect disease susceptibility and health during adult life. The present review aims at summarizing the current understanding of the intestinal epithelial innate immune system and its developmental and adaptive changes after birth.

## Innate Immune Receptor Expression by Intestinal Epithelial Cells

Epithelial cells line the surface of the intestinal mucosa. Together with the mucus layer, they generate the physical barrier between the largely sterile underlying tissue and the enteric lumen exposed to nutritional antigens, inhabited by a dense and dynamic microbiota and challenged by orally acquired pathogenic microorganisms. In addition, epithelial cells actively contribute to host-microbial homeostasis, antimicrobial host defense, and epithelial barrier repair. The presence of microbial organisms is detected by the expression of a variety of different innate immune receptors that survey the apical and basolateral plasma membrane, endosomal compartments, and the cytosol. Expression of members of the Toll-like receptor (TLR) family, such as TLR2, 3, 4, 5, and 9, the nucleotide-binding and oligomerization domain (NOD) receptor 1 and 2 and the helicases retinoic acid inducible gene (RIG-I), and the melanoma differentiation associated gene-5 (MDA5), initiate signal transduction cascades via the NF-κB pathway, mitogen activated protein (MAP) kinases, and interferon regulatory factors (IRFs), and influence epithelial gene expression (1–3). Inflammasome members such as the NOD receptor-related pyrin domain-containing NLRPs NLRP3, 6, and NLRC4 activate caspase 1 and facilitate the processing of preformed pro-IL-1β and pro-IL-18 and the release of bioactive cytokines as well as the induction of pyroptosis (4–7). In contrast, NLRP12 appears to inhibit canonical and non-canonical NF-κB signaling (8). Additionally, cell-autonomous mechanisms such as the formation of autophagosomes restrict microbial invasion and mucosal translocation in epithelial cells (9, 10). Recent reports have highlighted links between these different pathways. For example, NOD1 and 2 were shown to recruit autophagy-related protein 16-like 1 (ATG16L1) to the site of bacterial entry (11). Also, MyD88-dependent cell stimulation downstream of TLR or IL-1R stimulation was linked to autophagy-dependent antibacterial host defense in epithelial cells (9, 12). Although experimental evidence suggests the functional presence of innate immune receptors at the intestinal epithelium, a systematic analysis of epithelial cell specific receptor deficient animals has not been performed. The use of bone marrow chimeric mice on the other hand suffers from the potential radio-resistance of mucosal immune cells and the large diversity of stromal cells.

## Innate Immune Signaling during Ontogeny: Age-Dependent Receptor Expression and Downstream Signaling

**Figure 1:**
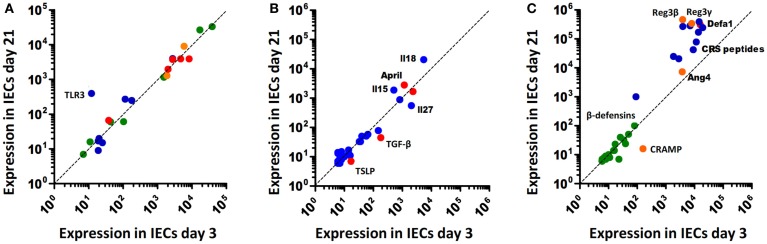
**Age-dependent expression of innate immune receptors, signaling, and effector molecules in murine intestinal epithelial cells**. **(A)** Innate immune receptors and inhibitory molecules. TLRs, blue; NLRs, green; RLRs, orange; negative regulators (SIGIRR, A20, PPARy, IRAK-M, IKK2), red. **(B)** Soluble intermediates: interleukins, blue; epithelial-derived modifiers of myeloid cells, red. **(C)** Antimicrobial effector molecules: (β-defensins, green; CRS peptides, blue; α-defensins (Defa), red; Reg3β/γ, Angiogenin4 (Ang4), and CRAMP, orange.

Recent reports suggest significant alterations of epithelial innate immune signaling during ontogeny, i.e., the transition from the protected environment *in utero* to the microbially and environmentally exposed life after birth. Although only beginning to be understood, the changes in the epithelial innate immune response after birth might significantly contribute to establish a stable life-long host-microbial homeostasis. On the other hand, it may predispose the neonate to certain infectious or inflammatory diseases. Indeed, many pathogens of the neonate host such as group B streptococci, *Listeria monocytogenes*, or *Escherichia coli* K1 rarely cause disease in adult individuals. Similarly, necrotizing enterocolitis (NEC), an inflammatory enteric disease of unknown etiology is largely restricted to the population of preterm human neonates. In mice, the observed postnatal alterations in epithelial innate immune signaling are accompanied by significant developmental changes. For example, crypts appear only during the second week after birth generating the niche for pluripotent Lgr5^+^ stem cells. Stem cells generate the rapidly proliferating pool of so-called transit-amplifying (TA) cells. Enterocyte proliferation within the crypts facilitates the continuous crypt-villus migration and rapid cell turnover in adult animals (13). In the absence of crypts, epithelial proliferation and renewal are markedly diminished in the neonate animal. Cell lines and primary epithelial cells isolated from fetal intestinal tissue express innate immune receptors, respond to microbial ligands, and secrete pro-inflammatory chemoattractants (14–17). Regulatory mechanisms must, therefore, exist to prevent inappropriate immune stimulation. Hackam and colleagues described downregulation of epithelial TLR4 expression and upregulation of epithelial TLR9 expression prior to birth in mice (18). Since TLR4 mediated epithelial signaling has been associated with mucosal damage (19) and TLR9 stimulation was suggested to dampen inflammation (20), this early adaptive regulation might prepare the fetal epithelium to microbial exposure during the immediate postnatal period. Similarly, our group showed that TLR3 expression in the neonate intestine in mice is low and increases only with weaning (Figure 1A) correlating with the enhanced susceptibility to rotavirus infection during the postnatal period (21). Since TLR3 was shown to amplify the antiviral response by upregulation of the helicases Rig-I and MDA5, low epithelial TLR3 expression in neonate mice might have a broader effect and indirectly also impair the helicase-mediated host response during the postnatal period despite unaltered basal helicase expression levels (22). In contrast to TLR3, members of the NOD-like receptor family, such as NOD1 and 2 but also the inflammasome constituents NLRP1, NLRP3, NALP6, and NALP12 as well as caspase 1 do not underlie any detectable developmental regulation on transcriptional level (Figure 1A). Enhanced expression of the negative regulatory molecules A20, single immunoglobulin IL-1 receptor-related molecule (SIGIRR), interleukin 1 receptor associated kinase (IRAK)-M, and Toll-interacting protein (TOLLIP) has been described in mature human neonatal epithelial cells (23). In mice, however, no major change in their expression level is observed (Figure 1A). Also, immune cell-mediated regulatory mechanisms such as neonatal B cell-derived IL-10 or arginase 2 secretion by newborn CD71^+^ erythroid cells may dampen the mucosal immune stimulation (24, 25). Finally, constituents of the amniotic fluid, colostrum, and breast milk were described to exhibit a negative regulatory effect on mucosal innate immune stimulation during the neonatal and pre-weaning period (26, 27). Thus, developmental and environmental mechanisms appear to restrict epithelial stimulation by innate immune receptors during the postnatal period.

On the other hand, exposure to environmental, nutritional, and microbial stimuli after birth appears to induce a protective epithelial response. For example, increased epithelial expression of the pro-inflammatory chemokine Cxcl2 and the NF-κB induced microRNA miR146-a were observed during the first hours after vaginal delivery in mice (15, 16). Also an increase of intestinal TNF mRNA levels was described after birth (28). In human beings, elevated calprotectin levels were measured in healthy term neonates during the first days after birth that reached concentrations comparable to patients with inflammatory bowel disease (29). This stimulation, however, is transient and appears not to be associated with mucosal damage or clinical disease. Postnatal immune activation induces adaptive negative regulatory mechanisms such as downregulation of the TLR signaling molecule IRAK1. Low epithelial IRAK1 protein expression prevents inappropriate transcriptional activation and epithelial cell damage. In addition, it drives a sustained epithelial expression profile that includes genes involved in metabolism, cell survival, and differentiation (16). IRAK1 is involved in the upstream signaling cascade of most TLRs. Only TLR3 signaling occurs completely independent of MyD88/IRAK1 *via* the adaptor molecule TIR-domain-containing adapter-inducing interferon-β (TRIF) (30). TLR3 expression, however, is significantly downregulated in the neonate intestine by the developmental transcriptional repressor B lymphocyte-induced maturation protein (Blimp)1 as described above (21, 31, 32). IRAK1 protein downregulation and low Tlr3 expression during the postnatal period might explain why deletion of A20, SIGIRR, or TOLLIP does not lead to spontaneous inflammation after birth (33–35). It may also cause the reduced pro-IL-18 expression in neonatal epithelial cells and thereby prevent excessive inflammasome-mediated tissue stimulation (Figure 1B). Thus, adaptive mechanisms in addition to the above discussed developmental mechanisms restrict epithelial innate immune activation in the homeostatic neonatal intestine. Following bacterial challenge with enteropathogenic microorganisms, however, the neonatal epithelium is able to respond in a MyD88-dependent fashion (own unpublished observation). The mechanisms that allow stimulation of the neonate epithelium in the event of immediate danger to mount a protective antimicrobial host response have not been investigated but may require contribution from immune cells in the *lamina propria*.

TLR, NLR, and helicase stimulation but also IL-1 and IL-18 signaling converge at the level of TGF-β activated kinase (TAK)1, inhibitor of κB kinase (IKK)1/2, and NF-κB essential modulator (NEMO) to induce NF-κB activation in epithelial cells. NF-κB signaling inhibits pro-apoptotic pathways and drives the expression of antimicrobial host defense mechanisms. A number of studies have highlighted the protective role of NF-κB mediated signaling at the epithelium during the postnatal period. For example, 10–15% of epithelial RelA deficient mice develop intestinal symptoms as early as 2–3 days after birth and succumb to mucosal bleeding both in the small and large intestine before weaning (36). Also, asymptomatic RelA deficient neonates exhibit reduced expression of anti-apoptotic and antimicrobial effector molecules. Similarly, enterocyte-specific TAK1 deficient mice develop spontaneous intestinal inflammation and epithelial apoptosis at the time of birth (28). Surprisingly, signs of epithelial apoptosis and inflammation were already observed prior to birth. Both TNF mediated cell stimulation and enhanced susceptibility to reactive oxygen species (ROS) cause mucosal damage in the neonate host (28, 37). Similarly, epithelial loss of NEMO or IKK1/2 expression results in mucosal bleeding, epithelial apoptosis, loss of mucosal integrity, and bacterial translocation and finally significant upregulation of pro-inflammatory cytokines during the first weeks after birth. Inflammation is driven by MyD88- and TNF-dependent pathways. Strikingly, loss of epithelial NEMO or IKK1/2 expression altered the colonic mucosa but left the small intestinal tissue largely unaffected (38). No clinical symptoms are observed during the postnatal period in mice expressing a dominant negative MyD88 transgene in the epithelium possibly due to remaining compensatory NF-κB activating pathways. Transgene animals initially gain weight indistinguishable from littermate controls but exhibit enhanced epithelial proliferation and develop spontaneous small intestinal inflammation at 24 weeks after birth (39). Epithelial-specific MyD88 deficient mice show an impaired mucosal barrier formation but no spontaneous inflammation after the postnatal period (40). Dysfunction in both animal models was at least in part explained by reduced expression of enteric antimicrobial peptides (AMPs). Similarly, MyD88-dependent signaling by radioresistant, non-myeloid cells has been shown to provide protection from inflammation in a chemically induced model of colitis *via* enhanced signaling through the epidermal growth factor receptor (EGFR) (41). In contrast to the strong phenotype of mice impaired in epithelial NF-κB signaling, epithelial-specific deletion of the mitogen activated protein (MAP) kinase p38 results in an only moderate phenotype. Decreased numbers of goblet cells and an enhanced rate of epithelial proliferation were noted in the adult colon in the absence of overt inflammation (42). In addition to NF-κB, also the alternative death complex protects from epithelial cell damage early after birth. Lack of intestinal epithelial FADD expression results in epithelial necroptosis with loss of small intestinal Paneth cells and spontaneous inflammation in small and large intestine associated with 50% mortality prior to weaning. Although the phenotype was rescued in the absence of Rip3 both in small and large intestine, only the colitis was dependent on MyD88 and TNF. Small intestinal inflammation might be the consequence of reduced antimicrobial peptide secretion (43). Thus, although excess immune activation may lead to organ damage, homeostatic innate immune stimulation appears to be required to prevent epithelial cell death, and drive antimicrobial host defense early after birth. The degree and nature of this homeostatic epithelial signaling may well vary during postnatal development and requires further investigations.

## “Cross-talk” between the Epithelium and Underlying Immune Cells

Epithelial chemokine secretion upon innate immune stimulation induces the recruitment of professional immune cells to help combating infection. Consistently, enhanced epithelial chemokine expression in adult transgene animals leads to increased recruitment of granulocytes and lymphocytes to the *lamina propria* (42). Epithelial signals also help to orchestrate adaptive immune functions. The secretion of thymic stromal lymphopoietin (TSLP) inhibits DC-derived IL-12 secretion and T_H_1 differentiation (44) and, thus, drives the T cell-mediated immune response towards T_H_2. A T_H_2-prone immune response with high IL-4, IL-5, IL-13, and IL-10 levels is typical for the neonate host within its anti-inflammatory environment (45). Epithelial TSLP production remains unaltered and the increasing demand for TSLP is provided by breast milk, as recently reported by MacFarlane *et al*. (46). TGF-β is another component of breast milk with anti-inflammatory properties (47). But, in contrast to TSLP, epithelial-specific expression of TGF-β is age-dependent and significantly more pronounced around birth (Figure 1B). In addition to retinoic acid and TSLP, TGF-β is a strong inducer of tolerogenic CD103^+^DCs, which, in turn, promote T_REG_ maturation (48). T_REG_ cells secrete large amounts of anti-inflammatory cytokines, such as Il-10 (49) and, thus, contribute to the T_H_2 biased immune response of the neonate. Furthermore, it is widely accepted that T_REG_ cells are directly involved in the development of a life-long protection against allergic disorders, such as asthma and hay fever (50, 51). Although, underlying mechanisms are still elusive, significantly higher levels of regulatory T cells and T_REG_-derived interferon (IFN)-γ were observed (52).

Epithelial-derived IL-25 recruits intraepithelial lymphocytes and IL-7 and trans-presented IL-15 maintains their presence (53). The expression of IL-15 is only enhanced after weaning (Figure 1B). An underlying reason is unknown but it can be speculated that expression of IL-15 and its trans-presenting receptor IL-15Rα by epithelial cells goes along with the development and appearance of its target cells, IL-2Rβ expressing intraepithelial intestinal lymphocytes (IELs). Similarly, the negligible epithelial secretion of a proliferation-inducing ligand (April) and B-cell-activating factor (BAFF) that facilitate T cell-independent IgA production (54) around birth (Figure 1B) most probably relies on the kinetic of B cell homing to the gut mucosa, which starts only after birth (55).

## Epithelial Innate Immune Effector Molecules

Protective epithelial effector functions include the synthesis of antimicrobial molecules, maintenance of the mucus layer, secretion of signaling intermediates, and an enhancement of epithelial tight junctions and cell turn over to remove infected or damaged cells. The active role of the epithelium appears to be critical also under homeostatic conditions to maintain barrier integrity. Epithelial-specific knockouts that impair cell signaling (28, 38, 39), disturb histone acetylation and microRNA function (56, 57), reduce apoptosis (43), alter epithelial cell differentiation (58, 59) or decrease mucus production (60), and lead to tissue damage and mucosal inflammation. Innate immune stimulation reinforces tight junction expression and regulates the epithelial barrier function (61). Also, epithelium-derived antimicrobial molecules such as Reg3γ, defensins or reactive oxygen, or nitrogen species provide direct antibacterial activity (40, 62). Effector molecules with antimicrobial and barrier promoting properties support the maintenance of gut homeostasis. The most abundant effector molecules in the intestine are AMPs (63). They significantly influence the microbial composition but also the susceptibility to infection (64). AMPs comprise members of different protein families, including defensins, defensin-like molecules such as cryptdin-related sequence (CRS) peptides, cathelicidins, C-type lectins, such as the regenerative islet-derived proteins 3 β and γ (Reg3β and Reg3γ), and RNAses, such as angiogenin 4 (Ang4) (65). Defensins are highly cationic peptides with an average length of approximately 30–40 amino acids. They encode for six cysteine residues that form characteristic, intramolecular disulfide bonds, and prevent peptide degradation (66, 67). Paneth cells in the small intestine constitute the main reservoir of α-defensins (68). β-defensins are mainly expressed in the colon by absorptive colonocytes. Whereas the genome of mice encodes more than 20 different α-defensins (also termed cryptdins in mice), human beings only produce two, human defensing (HD)-5 and HD-6 (63, 69). CRS peptides are evolutionary closely related to cryptdins and share a variety of properties such as the generation by Paneth cells as an inactive pro-form (69). In contrast to cryptdins, CRS peptides have the potential to form covalent homo- and heterodimers and, thus, are able to assemble a large number of different peptide molecules (70). The transcription of α-defensins and CRS peptides is largely constitutive and not subjective to known environmental signals (71). Release from Paneth cells, in contrast, occurs in response to microbial but also nervous and endogenous mediators (72, 73). In contrast to cryptdins, epithelial Reg3β and Reg3γ as well as Ang4 expression is MyD88 dependent and enhanced in the presence of the enteric microbiota or the pro-inflammatory response to environmental or infectious stimuli (40, 74). Significant differences in the antimicrobial peptide spectrum exist along the length of the small intestine but also between different mouse strains (75). This fact requires attention, since it has led to the misinterpretation of results by comparing, e.g., insufficiently backcrossed gene animals in the past (76).

## The Antimicrobial Peptide Repertoire during Ontogeny

The neonate small intestinal epithelium expresses the cathelicidin cathelin-related antimicrobial peptide (CRAMP) (77). CRAMP exerts antibacterial activity against commensal and pathogenic bacteria *in vitro* and protects the neonate intestinal mucosa from *Listeria monocytogenes*, a leading cause of neonatal sepsis and meningitis in newborns. Production of Paneth cell-derived AMPs like cryptdins and CRS peptides only starts approximately day 9 *post partum* in C57BL/6 mice as shown in Figure 1C, reflecting the delayed appearance of small intestinal Paneth cells during the postnatal period (77, 78). Since intestinal epithelial CRAMP expression wanes after the postnatal period this results in a switch in the peptide repertoire and production site from epithelial CRAMP expression in the neonate to Paneth cell-secreted cryptdins and CRS peptides after weaning (Figure 1C). Adult mice deficient for the metalloproteinase (MMP)-7, and, therefore, unable to generate mature cryptdins and CRS peptides show a higher number of Firmicutes and a lower amount of Bacteroidetes in the small intestine (64). Since the transition in nutrition from breast milk to solid food goes along with a reduction of Firmicutes, the specific antimicrobial potential of cryptdins might help to prepare the neonatal intestine for weaning. Also, the lack of Paneth cell-derived defensins in the neonate host might explain the susceptibility of neonate but not adult mice to oral challenge with *Shigella flexneri* (79, 80). Consistently, mice lacking MMP-7 are more susceptible to invasive *Salmonella* Typhimurium infection and exhibit a reduced ability to clear the bacteria from the small intestine (81). The expression of Reg3β/γ, and Ang4 is maximal in adult animals (Figure 1C). This may in part be attributable to the low stimulatory potential of the developing enteric microbiota and facilitate early colonization by mainly gram-positive commensal bacteria (82). Importantly, AMPs in adult mice act in concert with the mucus layer overlaying the epithelium both in the small and large intestine. Release by Paneth cells leads to enrichment of cryptdins and CRS peptides in mucus material generating a physico-chemical shield to protect the epithelial surface (83, 84). Similarly, also Reg3γ acts to promote the mucus barrier and Reg3β/γ and Ang4 expression was noted in mucus-producing goblet cells (62, 85). The neonate intestine displays a reduced mucus layer [(86) and own unpublished observation]. Expression of CRAMP by enterocytes lining the epithelial surface might compensate for the reduced mucus layer to provide protection of the epithelial surface.

**Figure 2:**
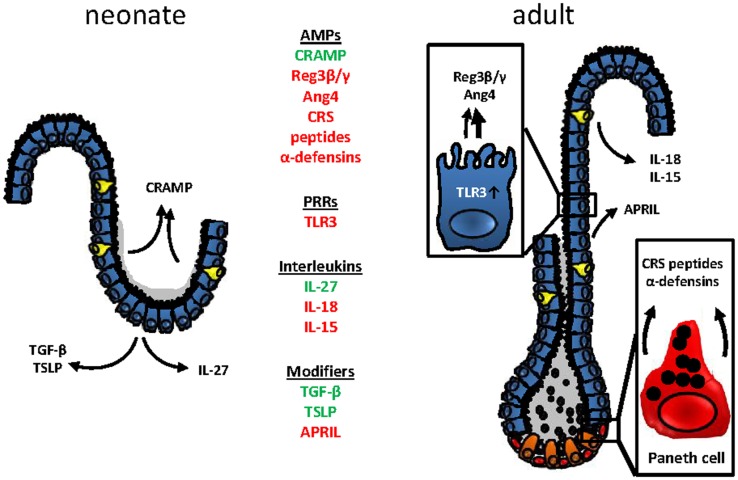
**Age-dependent innate immune receptor and effector molecules expression at the mouse intestinal epithelium**. Changes in the epithelial architecture between the murine neonate (left) and adult (right) intestinal epithelium. Age-dependently expressed genes are highlighted in green (upregulation in the neonate epithelium) or red (downregulated in the neonate epithelium).

In conclusion, developmental and adaptive changes accompany the establishment of mucosal host-microbial homeostasis in the intestine after birth (Figure 2). Although the functional importance of these changes is incompletely understood, the observed mechanisms appear to contribute to the transition from the protected environment *in utero* to the microbially and environmentally exposed life after birth. A deeper understanding requires further investigations but might ultimately help to explain the development of inflammatory but also metabolic diseases and allow the development of prophylactic and therapeutic strategies.

## Conflict of Interest Statement

The authors declare that the research was conducted in the absence of any commercial or financial relationships that could be construed as a potential conflict of interest.
